# PET/CT Volumetric Parameters as Predictors of the Peritoneal Cancer Index in Advanced Ovarian Cancer Patients

**DOI:** 10.3390/diagnostics15141818

**Published:** 2025-07-19

**Authors:** Ariel Glickman, Blanca Gil-Ibáñez, Aida Niñerola-Baizán, Marta Tormo, Núria Carreras-Dieguez, Pere Fusté, Marta Del Pino, Eduardo González-Bosquet, Inmaculada Romero-Zayas, Cristina Celada-Castro, Tiermes Marina, Lydia Gaba, Adela Rodriguez Hernández, Adela Saco, Laura Buñesch, Josep Lluís Carrasco, Katherine Quintero, David Fuster, Berta Díaz-Feijóo, Aureli Torné, Pilar Paredes

**Affiliations:** 1 Gynecologic Oncology Unit, Institut Clínic de Ginecologia, Obstetrícia i Neonatologia, Hospital Clínic Barcelona, 08036 Barcelona, Spain; ncarreras@clinic.cat (N.C.-D.); pfuste@clinic.cat (P.F.); mdelpino@clinic.cat (M.D.P.); edugonzalez@sjdhospitalbarcelona.org (E.G.-B.); celada@clinic.cat (C.C.-C.); marina@clinic.cat (T.M.); bdiazfe@clinic.cat (B.D.-F.); atorne@clinic.cat (A.T.); 2 Facultat de Medicina i Ciències de la Salut, Universitat de Barcelona (UB), 08036 Barcelona, Spain; ninerola@clinic.cat (A.N.-B.); dfuster@clinic.cat (D.F.); 3 Gynecologic Oncology and Minimally Invasive Gynecologic Surgery Unit, Department of Obstetrics and Gynecology, 12 de Octubre University Hospital, 28041 Madrid, Spain; blanca.gil@salud.madrid.org; 4 Institut d’Investigacions Biomèdiques August Pi i Sunyer (IDIBAPS), 08036 Barcelona, Spain; lgaba@clinic.cat (L.G.); adrodriguez2@clinic.cat (A.R.H.); masaco@clinic.cat (A.S.); 5 Nuclear Medicine Department, Hospital Clínic Barcelona, 08036 Barcelona, Spain; tormo@clinic.cat (M.T.); icromero@clinic.cat (I.R.-Z.); kquintero@clinic.cat (K.Q.); 6 Biomedical Research Networking Center of Bioengineering, Biomaterials and Nanomedicine (CIBER-BBN), Instituto de Salud Carlos III, 28029 Barcelona, Spain; 7 Cancer Institute and Blood Diseases, Hospital Clínic Barcelona, 08036 Barcelona, Spain; 8 Pathology Department, Hospital Clínic Barcelona, 08036 Barcelona, Spain; 9 Radiology Department, Hospital Clínic Barcelona, 08036 Barcelona, Spain; lbunesch@clinic.cat; 10 Biostatistics, Department of Basic Clinical Practice, University of Barcelona, 08036 Barcelona, Spain; jlcarrasco@ub.edu

**Keywords:** FDG PET/CT, volumetric parameters, MTV, TLG, advanced ovarian cancer, peritoneal carcinomatosis

## Abstract

**Background:** Assessment of the peritoneal cancer burden is crucial for determining the optimal treatment in advanced ovarian cancer (AOC). Effective non-invasive methods to predict tumour load remain limited. This study aimed to assess the applicability of 2-[^18^F]FDG PET/CT volumetric parameters, metabolic tumour volume (MTV), and total lesion glycolysis (TLG) for predicting the surgical peritoneal cancer index (PCI) in AOC before primary treatment. **Methods:** Patients with high-grade serous or undifferentiated AOC who underwent surgical PCI evaluation and 2-[^18^F]FDG PET/CT between 01/2013 and 12/2018 were included. MTV and TLG were calculated using thresholds of 40% and 50% (MTV40, MTV50, TLG40, and TLG50). Correlations between the peritoneal carcinomatosis MTV (car_MTV) and TLG (car_TLG) were analysed. The capacity of volumetric parameters to estimate PCIs above or below 14 and 20 was assessed for the whole abdominal cavity and in per-quadrant analysis, specifically for upper-abdomen areas 1, 2, and 3 (MTV40_1, 2, 3 and TLG40_1, 2, 3). **Results:** MTV40, MTV50, TLG40, and TLG50 significantly correlated with the PCI in the final study population (*n* = 45). MTV40 showed a Pearson coefficient of 0.41 (*p* = 0.003). MTV3_40 (AUC 0.79) and TLG3_40 (AUC 0.81) presented the highest AUCs for predicting a PCI above or below 14. The volumetric parameters allowed the prediction of a PCI greater or less than 20, with an AUC of 0.77 for MTV40_1 and 0.78 for TLG40_1. **Conclusions:** 2-[^18^F]FDG PET/CT MTV and TLG correlate significantly with the surgical PCI when assessing peritoneal carcinomatosis or quadrant-specific disease. This approach offers a reliable non-invasive method for evaluating tumour burden in AOC.

## 1. Introduction

Ovarian cancer is the most lethal of all gynaecological malignancies, with epithelial ovarian carcinoma representing approximately 90% of the diagnoses [1]. High-grade serous ovarian carcinoma (HGSOC) and undifferentiated ovarian carcinoma (UOC) account for more than 80% of cases. Nearly 70% of patients present with advanced ovarian cancer (AOC) (International Federation of Gynaecology and Obstetrics [FIGO] stage III–IV) at the time of diagnosis, with a five-year survival rate of less than 30% [2].

Despite advances in adjuvant treatment, upfront primary debulking surgery (PDS) of all macroscopic disease, followed by platinum-based chemotherapy, remains the standard of care, offering the best survival rates [3]. Neoadjuvant chemotherapy (NACT) and interval debulking surgery are recommended when complete cytoreduction is not possible. Accurate assessment of the extent and distribution of the disease is crucial for selecting the most adequate treatment strategy.

While FIGO staging is widely used, it offers limited insight into the total disease burden, implant distribution, and the feasibility of cytoreduction in AOC patients. The estimation of peritoneal tumour burden remains challenging, and surgical evaluation via laparoscopy or laparotomy is considered the gold standard for predicting complete resection [4]. The peritoneal cancer index (PCI), introduced by Jacquet and Sugarbaker [5], describes the tumour load and anatomical distribution of peritoneal carcinomatosis. It is the most universally validated tool for assessing intra-abdominal disease burden by surgical evaluation [6,7,8,9]. The prognostic value of the PCI in AOC patients is well established [10,11], with scores below 14 correlating with high rates of complete cytoreduction, whereas scores above 20 are associated with a greater likelihood of suboptimal surgery [11,12]. Currently, there are no validated non-invasive methods that allow robust prediction of the surgical PCI.

Standard non-invasive methods, such as serum tumour markers and abdominal computed tomography (CT), are used in clinical practice to evaluate tumour load before primary treatment [3,13]. While CT scans offer valuable anatomical details, they can overlook critical locations and lack volumetric or metabolic information regarding peritoneal carcinomatosis, the most frequent clinical presentation in AOC patients.

Positron emission tomography (PET)/CT with 2-[^18^F]fluoro-2-deoxy-D-glucose (2-[^18^F]FDG), hereafter referred to as 2-[^18^F]FDG PET/CT, provides metabolic information on the behaviour of ovarian cancer and allows the assessment of tumour burden [14,15]. It offers an overview of tumour spread into the peritoneal cavity and beyond, significantly impacting staging and treatment in AOC recurrence [16]. The standardised uptake value (SUV), particularly the highest metabolic activity within an area of interest (SUVmax), is commonly used to quantify metabolic activity and estimate tumour burden [17]. However, volumetric parameters, such as metabolic tumour volume (MTV) and total lesion glycolysis (TLG), assess the metabolic activity of the entire tumour and take into account the volume of the disease [15]. They have been shown to reflect tumour burden better than SUVmax [18], and their utility as prognostic factors has been demonstrated in ovarian cancer patients [19]. While MTV measures the volume of a tumour with increased tracer uptake, TLG quantifies the sum of metabolic activity within the MTV [20].

Both the PCI and 2-[^18^F]FDG PET/CT have proven to be important prognostic factors in AOC [21,22]. However, the correlation between the intraperitoneal tumour burden determined by PCI and PET/CT volumetric parameters (MTV and TLG) has rarely been explored [20,21,22,23]. This study aimed to assess the clinical applicability of MTV and TLG in predicting the PCI obtained through surgery by evaluating their correlation in patients with advanced-stage HGSOC or UOC, prior to primary treatment.

## 2. Materials and Methods

### 2.1. Study Design and Participants

The study included patients with AOC referred to the Gynaecological Oncology Unit at the Hospital Clínic of Barcelona between January 2013 and December 2018. To ensure the homogeneity of the cohort, the inclusion criteria were as follows: (1) newly diagnosed HGSOC or UOC; (2) FIGO stage III-IV; (2) diagnostic surgical evaluation (laparoscopy or laparotomy); and (3) 2-[^18^F]FDG PET/CT performed before primary treatment. The exclusion criteria were as follows: (1) diagnosis of another synchronous tumour; (2) pregnancy; (3) unresectable stage IV disease eligible for NACT with a prior biopsy; and (4) PET/CT performed at another centre.

Before primary treatment, the concentrations of the tumour markers CA125 and human epididymis protein 4 (HE4) and serum creatinine (sCr) were obtained in all the subjects. Abdominal CT, gynaecological ultrasound, and 2-[^18^F]FDG PET/CT were performed prior to a surgical procedure to estimate the PCI. Demographic and clinical data were collected from patient records. This retrospective study was approved by the institutional Ethics Committee of the Hospital Clínic of Barcelona (registry number HCB/2019/0215). Written informed consent was obtained from all the subjects.

### 2.2. Peritoneal Cancer Index Assessment by Surgery

Patients underwent a surgical diagnostic procedure to evaluate the extension of the disease, confirm imaging findings, obtain samples for histology, and calculate the PCI. To obtain the PCI [5], the peritoneal cavity was divided into 13 abdominopelvic regions using four lines: two vertical midclavicular lines and two horizontal lines passing through the anterior superior iliac spine and under the costal arcs. This division results in 9 areas (0–8), with the upper and lower jejunum and ileum constituting the remaining 4 areas. The PCI assesses the distribution and volume of peritoneal carcinomatosis by grading each region from 1 to 3 based on the size of the largest tumour: 1 for implants smaller than 5 mm, 2 for implants from 5 mm to 5 cm, and 3 for disease greater than 5 cm or confluent disease. The total score was calculated by summing the scores of each region, with 39 being the maximum possible score. For analysis, the PCI was categorised into two levels: 1–13 or ≥14 and 1–19 or ≥20 [10,24].

### 2.3. 2-[^18^F]FDG PET/CT

**Figure 1:**
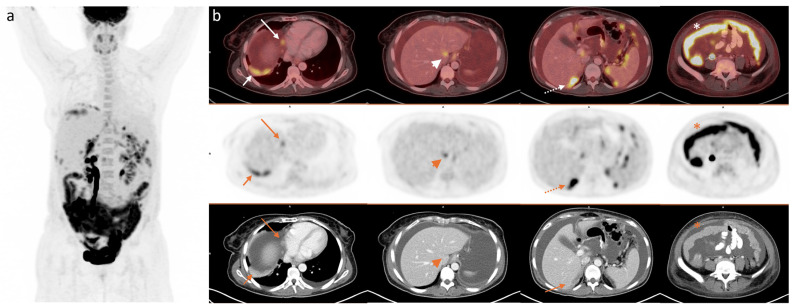
2-[^18^F]FDG PET/CT scan of a 68-year-old woman with AOC prior to first-line treatment showing (**a**) maximum intensity projection (MIP) and (**b**) axial images of fused PET/CT (upper), PET (middle), and CT (lower row) with peri-hepatic carcinomatosis (arrows), liver hilum disease (head arrow), Morison pouch implant (dotted arrow), and omental cake (*).

Whole-body 2-[^18^F]FDG PET/CT scans were performed using a Biograph mCT TrueV PET/CT hybrid device (Siemens Healthineers, Erlangen, Germany) fitted with time-of-flight (TOF) technology and a 64-slice Somatom Definition spiral CT for attenuation correction and image fusion (Figure 1). Images were acquired following the European Association of Nuclear Medicine guidelines [25,26]. After a 6-h fasting period to achieve blood glucose concentrations below 180 mg/L, patients were asked to rest quietly in a dimly lit room for 60 min after intravenous administration of 4.07 MBq/kg (0.11 mCi/kg) of 2-[^18^F]FDG. PET/CT images were acquired from the base of the skull to the proximal third of the thighs with the arms raised above the head. The PET images were acquired in 3D mode, with acquisition lasting 2.5 min per bed position. The chest sections were imaged during shallow breathing. The parameters for CT acquisition were as follows: 100 kV, 120 mA (with the CARE dose system), 5 mm slice thickness, duration time of 0.5, and pitch of 0.8.

An ionic iodinated solution with an iodine concentration of 370 mg/mL (Gastrografin^®^, Bayer AG, Leverkusen, Germany) was used for oral contrast. A total of 3.7 g per patient, distributed in two intakes, was administered: 5 mL of solution at the time of 2-[^18^F] FDG injection and 5 mL just before acquisition. PET data reconstruction was performed via the iterative algorithm True X + TOF (ultra-HD − PET), which applies attenuation and point spread function correction in addition to the commonly used correction factors. A Gaussian filter of 2 mm was applied, and 2 iterations/21 subsets, zoom 1, and a 200 × 200 matrix were used. For the CT data, soft-tissue reconstruction was used (B30f homogeneous medium) for the whole-body image, and the B60f defined was used for lung reconstruction.

### 2.4. Image Analysis

For the assessment of tumour burden, any lesion ≥2 mm exhibiting a higher uptake than surrounding tissues was considered tumoural disease. Two nuclear medicine specialists independently reviewed the images, and volumes of interest (VOIs) were delineated by consensus.

**Figure 2:**
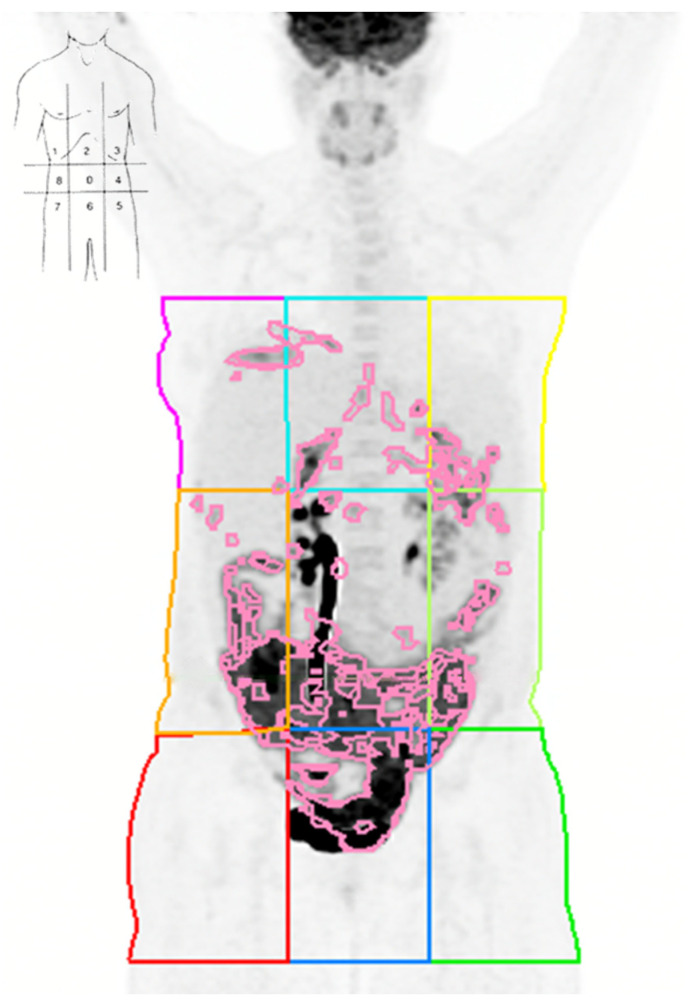
FDG-PET/CT maximum intensity projection (MIP) of a patient with AOC showing how images were subdivided into nine PCI regions.

Spherical or ellipsoidal VOIs were delimited in every pathological uptake in the PET images with the correction of attenuation using an MIM Vista Workstation (version 7.3.6, MIM Software Inc., Cleveland, OH, USA), ensuring the inclusion of all hypermetabolic lesions in axial, sagittal, and coronal projections. The maximum and mean SUVs were automatically calculated on the basis of the measured activity concentration (Bq/mL) multiplied by the patient’s weight (kg) divided by the injected activity (Bq). MTV and TLG values were calculated using SUVmax thresholds of 40% (MTV40, TLG40) and 50% (MTV50, TLG50) [19]. The peritoneal carcinomatosis MTV (car_MTV) and TLG (car_TLG) were computed for the abdominal cavity, excluding the retroperitoneal nodes, solid organs, and distant metastases. PET/CT images were partitioned into nine regions based on the anatomical boundaries described by Sugarbaker [5] (Figure 2). MTV and TLG were subsequently calculated individually for each of these regions (quadrants 0 to 8). The analysis focused on the predictive capabilities of volumetric parameters corresponding to the entire abdominal cavity and, for the per-quadrant analysis, focused on areas related to the upper abdomen (areas 1–2–3) to concentrate on this clinically relevant area and avoid intrinsic biases.

### 2.5. Statistical Analysis

The description of quantitative variables was carried out by computing the mean and standard deviation (SD) if the assumption of normality was met; otherwise, the median and interquartile range (IQR) were used. Counts and percentages were used for categorical variables. The Mann–Whitney test was used to compare the values of PET/CT volumetric parameters between groups, defined in terms of a PCI less and greater than or equal to thresholds of 14 and 20. The diagnostic ability of volumetric parameters to predict PCI status was assessed by the area under the receiver operating characteristic (ROC) curve, using binormal smoothing to reduce variability due to the limited sample size. Diagnostic thresholds were computed by maximising the Youden index [27]; sensitivity and specificity were also computed. The significance of the tests was evaluated using a type-I error rate of 5%.

## 3. Results

### 3.1. Patient Selection and Baseline Characteristics

**Figure 3:**
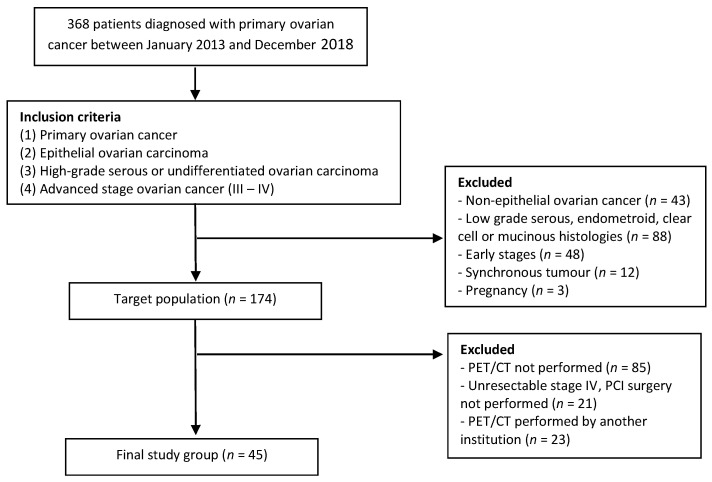
Flowchart showing the inclusion and exclusion criteria of the final study cohort.

During the study period, 368 patients were diagnosed with primary ovarian cancer. A flowchart showing the final study population of 45 patients after applying the inclusion and exclusion criteria is presented in Figure 3. Forty patients (88.9%) presented with HGSOC, and five presented with UOC (11.1%). The mean age at diagnosis was 61.9 years (SD = 10.9). The median sCr value was 0.73 mg/dL (IQR [0.22]). The median values for CA125 and HE4 were 469 IQR [1724] and 456 IQR [1212], respectively. Table 1 shows the characteristics of the patients.

### 3.2. PET/CT Parameters, Global Tumour Burden, and Surgical PCI

Diagnostic surgery was performed via laparoscopy in 29 patients (64%) and laparotomy in 16 patients (36%). The median PCI was 17 IQR [9]. Fifteen patients (33.3%) were selected for PDS and thirty (66.7%) were selected for NACT, with mean PCIs of 12.79 (SD = 6) and 18.17 (SD = 7), respectively. Significant differences were found between the two groups (*p* = 0.02). The standard and volumetric parameters of whole-body and intraperitoneal 2-[^18^F]FDG PET/CT are shown in Table 2.

The intraperitoneal tumour load corresponding to peritoneal carcinomatosis assessed by MTV using thresholds of 40% and 50% (car_MTV40, car_MTV50, car_TLG40, and car_TLG50) was significantly correlated with the surgical PCI. The strongest correlations were observed for car_MTV40 and car_MTV50 with Pearson’s coefficients of 0.41 (*p* = 0.003) and 0.38 (*p* = 0.013), respectively. TLG showed significant, albeit weaker, correlations: 0.32 (*p* = 0.015) for car_TLG40 and 0.33 (*p* = 0.034) for car_TLG50. Experimental data illustrating this are provided in Supplementary Figure S1.

### 3.3. Volumetric Parameters and PCI Stratification Thresholds

**Figure 4:**
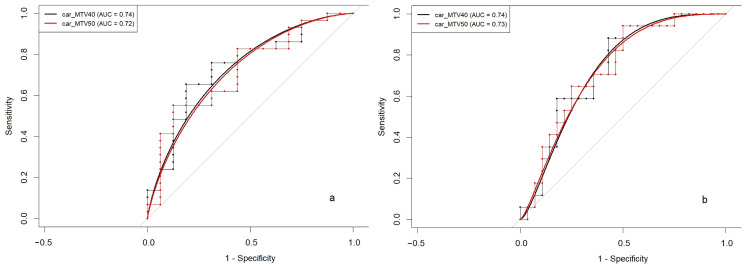
Receiver operating characteristic curves depicting the efficacy of car_MTV40 and car_MTV50 in the prediction of PCIs either above or below 14 (**a**), with respective AUC values of 0.74 and 0.72, and PCIs above or below 20 (**b**), with corresponding AUC values of 0.74 and 0.73. The sensitivity and specificity pairs used to generate the curves are shown on each graph. MTV40: metabolic tumour volume calculated with a 40% SUVmax threshold; MTV50: MTV calculated with a 50% SUVmax threshold; car: peritoneal carcinomatosis; PCI: peritoneal carcinomatosis index; AUC: area under the curve.

Significant differences were found between the MTV corresponding to patients with a PCI above and below 14 and 20, as shown in Table 3. Differences were also observed with TLG for the corresponding subgroups, but these did not reach statistical significance. The diagnostic potential of car_MTV40 and car_MTV50 for predicting a PCI higher or lower than 14 was evaluated, yielding area under the curve (AUC) values of 0.74 and 0.72, respectively (Figure 4a). The optimal cut-off points were determined. Through the use of 213.34 as the cut-off for car_MTV40 and 130.68 for car_MTV50, PCIs above or below 14 were predicted with sensitivities of 66% and 55% and specificities of 75% and 69%, respectively.

The efficacy in predicting a PCI greater or less than 20 showed an AUC of 0.74 for car_MTV40 and 0.73 for car_MTV50, as depicted in Figure 4b. This underscored the comparable predictive capacity of MTV in these subsets, in alignment with findings for a PCI threshold of 14. Consequently, specific cut-off values were determined: 240.79 for car_MTV40 and 147.35 for car_MTV50. These thresholds enabled the prediction of a PCI surpassing or remaining below 20, achieving sensitivities of 59% for both parameters and specificities of 68% and 75%, respectively.

### 3.4. Quadrant-Level Volumetric Parameters and PCI: Correlation and Diagnostic Performance

Both MTV40 and TLG40 corresponding to quadrants 1, 2 and 3 individually showed significant correlation with the PCI. The most notable correlations were found for MTV40_1 and TLG40_1, which presented Spearman correlation coefficients of 0.582 (*p* < 0.001) and 0.596 (*p* < 0.001), respectively. The correlations between the PCI and the volumetric parameters corresponding to quadrants 1, 2 and 3 in conjunction (MTV40_123 and TLG40_123) were significant (*p* < 0.001), with Spearman’s coefficients of 0.511 and 0.549, respectively. Table 4 shows all the correlations explored. The corresponding scatter plots showing the experimental data are presented in Supplementary Figure S2.

Regarding the per quadrant analysis, the highest AUC for predicting a PCI above or below 14 was obtained for MTV3_40 (AUC 0.79) and TLG3_40 (AUC 0.81). When the designed cut-offs of 2.5 and 5.95 were applied, the PCI could be predicted with sensitivities of 66% and 79% and specificities of 75% and 69%, respectively. Volumetric parameters of the upper abdomen also predicted a PCI greater or less than 20: the highest AUCs were obtained for MTV40_1 and TLG40_1 (0.77 and 0.78, respectively). These parameters showed sensitivities of 65% and 71% and specificities of 75% and 79%, respectively, with corresponding cut-off values of 2.96 and 6.27.

Finally, within the PDS subgroup, the Spearman correlation coefficients for the PCI and MTV40_1 and TLG40_1 were 0.633 (*p* = 0.011) and 0.678 (*p* = 0.005), respectively. When quadrants 1, 2 and 3 were considered together, the correlation coefficients were 0.766 (*p* = 0.001) for MTV40_123 and 0.791 (*p* < 0.001) for TLG40_123. These higher and statistically significant correlations are also shown in Table 4.

## 4. Discussion

The present study revealed a significant correlation between the volumetric parameters derived from PET/CT and the PCI obtained by surgical assessment in patients with AOC. This correlation suggests that MTV and TLG can potentially serve as non-invasive tools for assessing disease burden. To the best of our knowledge, this is the first study correlating the surgical PCI with continuous MTV and TLG variables, analysing both the entire abdominal cavity and specific quadrants.

Accurate preoperative assessment of disease dissemination is essential for determining the most appropriate treatment strategy for AOC. Traditionally, invasive procedures have been required to calculate the PCI via surgical evaluation [8]. However, non-invasive methods such as 2-[^18^F]FDG PET/CT are being explored, with MTV and TLG emerging as promising tools that provide objective information on disease activity and extension [23,28]. Although various formulas have been suggested for calculating SUV thresholds to derive MTV and TLG [15], thresholds set at 40% and 50% have shown superior outcomes in AOC patients. Our study revealed slightly better correlations between the PCI and MTV when a 40% threshold and its corresponding TLG were used. In accordance with previous data, there were no significant differences between these parameters when they were calculated using a 50% threshold [14]. Previous studies highlighted an enhanced clinical significance of MTV over TLG [29,30], which could be attributed to its non-threshold nature [19]. Nevertheless, the present findings did not support the greater reliability of MTV as an indicator of tumour burden in AOC when compared to TLG.

However, evidence on the correlation between PET/CT volumetric parameters and the PCI in patients with ovarian cancer is limited. Mallet et al. reported a significant correlation between the PCI and MTV40 (Spearman coefficient = 0.380, *p* < 0.001) and TLG40 (Spearman’s coefficient = 0.252, *p* = 0.021) in a study including 84 patients with AOC of various histologic subtypes (serous, endometrioid, clear cell, and mucinous) who underwent PET/CT and staging laparoscopy before primary treatment [18]. Our study, which focused on a more selective cohort of HGSOC and UOC patients, yielded slightly better results, with correlation coefficients of 0.41 and 0.33, respectively.

Numerous studies have investigated the ability of PET/CT to detect the presence of peritoneal implants to more accurately ensure patient selection for initial cytoreductive surgery. The use of 2-[^18^F]FDG PET/CT has demonstrated sensitivity ranging from 78.6% to 96.2% and specificity ranging from 91% to 92%. These values surpass the sensitivity of 53.6% to 88.5% and the specificity of 61% to 65% achieved with CT alone [31,32]. Some authors have even explored the capacity of PET/CT to perform per-quadrant analysis [18,33]. Nevertheless, determining only the presence of disease can hardly lead to decision-making. Additionally, prior investigations have retrospectively examined the utility of the per-quadrant PCI, using data acquired during primary surgery, to predict complete resection and survival outcomes in patients with AOC. These studies highlight the significant role that per-area disease can play in guiding treatment decisions [22].

The ability of PET/CT to differentiate small-bowel implants, which might be included in the middle (quadrants 8–0–4) and lower (quadrants 5–6–7) PCI regions, remains challenging [18,28,34,35]. Various strategies have been employed to address this issue, but none have consistently overcome the limitations of this technique [28,34,35,36,37]. The current study focused on the upper abdomen (areas 1–2–3) for per-quadrant analysis, revealing significant correlations between quadrant-specific MTV40 and TLG40 values and the PCI, particularly in quadrant 1. The combined values for quadrants 1, 2, and 3 (MTV40_123 and TLG40_123) also strongly correlated with the PCI, indicating the value of the upper abdomen for the prediction of overall disease involvement.

The predictive power of volumetric parameters to determine whether a PCI would surpass or remain below the clinically relevant thresholds of 14 and 20 was also explored. The robust AUCs for car_MTV40 and car_MTV50 show their potential as valuable tools for risk stratification. The calculated cut-off points show high sensitivity and specificity, underscoring their clinical applicability.

In the subgroup analysis of patients who underwent PDS, significant correlations were observed between metabolic volumetric parameters and the PCI in specific quadrants. This might be due to the reduced extent of carcinomatosis in the PDS subgroup, allowing for more precise identification and characterisation of implants [25]. Replicating these findings in larger series could enable the simultaneous scheduling of diagnostic laparoscopy and cytoreductive surgery.

The strengths of this study include its comprehensive evaluation of a clinically relevant cohort and exploration of volumetric PET/CT parameters in relation to surgical information such as the PCI. The homogeneous cohort, which focuses on high-grade tumours, and independent evaluation by two senior nuclear medicine specialists add to its robustness.

Based on our findings and the current workflow implemented in our institution, a structured diagnostic and surgical decision-making pipeline could be considered for patients with suspected advanced ovarian cancer. Following clinical evaluation, tumour marker analysis (e.g., CA-125, HE4), and the acquisition of 2-[^18^F]FDG PET/CT, the car_MTV40 value may aid patient stratification. Patients with a car_MTV40 below 213 cm^3^ (corresponding to an expected PCI below 14) may be considered for same-day diagnostic laparoscopy and PDS, provided that they are surgically fit. Those with intermediate values (213–240.79 cm^3^, potentially predicting a PCI between 14 and 20) could be discussed by a multidisciplinary tumour board to determine the suitability of a one-step or staged two-step approach. For patients with a car_MTV40 above 240.79 cm^3^ (presumably corresponding to a PCI greater than 20), diagnostic laparoscopy without planned immediate PDS may be advisable, as a high tumour burden could indicate poor resectability and the potential need for NACT. This volumetric-guided stratification is exploratory and should always be interpreted within the broader context of clinical judgement, imaging quality, and patient-specific considerations. Given the retrospective, single-centre nature of our study and the relatively small sample size, further prospective validation is warranted before routine clinical implementation.

## 5. Conclusions

FDG PET/CT-derived volumetric parameters demonstrate significant potential in evaluating peritoneal carcinomatosis and predicting disease burden in AOC patients. MTV and TLG offer a non-invasive alternative for estimating the surgical PCI and can help in first-line treatment decisions. However, large-scale, prospective, multicentre studies are essential to validate these findings before their routine clinical implementation.

## Figures and Tables

**Table 1:** Patient characteristics.

*n* = 45	Mean/Median	(SD)/[IQR]/%
Age (years)	61.9	(SD = 10.9)
Histology		
HGSOC	40 ˙	(88.9%)
UOC	5 ˙	(11.1%)
CA125 (U/mL)	469 *	[1724]
HE4 (pmol/L)	456 *	[1212]
sCr (mg/dL)	0.73 *	[0.22]
FIGO stage		
III	23 ˙	(51.1%)
IV	22 ˙	(48.9%)
PCI	17 *	[9]
Primary treatment		
PDS	15 ˙	(33.3%)
NACT	30 ˙	(66.7%)

IQR: Interquartile range; SD: standard deviation; HGSOC: high-grade serous ovarian carcinoma; UOC: undifferentiated ovarian carcinoma; sCr: serum creatinine; PCI: peritoneal carcinomatosis index; PDS: primary debulking surgery; NACT: neoadjuvant chemotherapy; *n* * mean; *n* ˙ median.

**Table 2:** 2-[^18^F]FDG PET/CT parameters.

*n* = 45	Mean	SD
SUVmax	18.4	8
SUVmean	7.84	4.01
wb_MTV40	239	137
wb_MTV50	147	88.4
wb_TLG40	1757	1057
wb_TLG50	1154	678
car_MTV40	229	141
car_MTV50	140	90.6
car_TLG40	1687	1097
car_TLG50	1101	704

SUV: standardized uptake value; max: maximum; mean: average; MTV40: metabolic tumour volume calculated with the 40% SUVmax threshold; MTV50: metabolic tumour volume calculated with the 50% SUVmax threshold; TLG40: total lesion glycolysis calculated using MTV40; TLG50: total lesion glycolysis calculated using MTV50; wb: whole-body; car: carcinomatosis (peritoneal dissemination); SD: standard deviation.

**Table 3:** 2-[^18^F]FDG PET/CT volumetric parameters and the peritoneal carcinomatosis index.

PCI Groups	<14 (*n* = 16)	≥14 (*n* = 29)	*p*	<20 (*n* = 28)	≥20 (*n* = 17)	*p*
car_MTV40	161 (118)	266 (140)	0.011	191(139)	291 (124)	0.016
car_MTV50	99 (74.4)	162 (92.0)	0.016	117 (89.7)	178 (80.7)	0.023
car_TLG40	1322 (936)	1888 (1142)	0.081	1506 (1097)	1985 (1063)	0.156
car_TLG50	864 (592)	1231 (737)	0.077	966 (655)	1322 (746)	0.115

MTV40: MTV calculated with a 40% SUVmax threshold; MTV50: MTV calculated with a 50% SUVmax threshold; TLG40: TLG calculated using MTV40; TLG50: TLG calculated using MTV 50%; car: peritoneal carcinomatosis; PCI: peritoneal carcinomatosis index.

**Table 4:** Correlations between metabolic tumour volume and total lesion glycolysis per quadrant and the surgical peritoneal carcinomatosis index.

Parameters	PCI	
	r *	*p*
All patients (*n* = 45)		
MTV40_1	0.582	<0.001
TGL40_1	0.596	<0.001
MTV40_2	0.420	0.004
TGL40_2	0.459	0.002
MTV40_3	0.418	0.004
TGL40_3	0.464	0.001
MTV40_123	0.511	<0.001
TGL40_123	0.549	<0.001
PDS patients (*n* = 15)		
MTV40_1	0.633	0.011
TGL40_1	0.678	0.005
MTV40_2	0.595	0.019
TGL40_2	0.535	0.040
MTV40_3	0.603	0.017
TGL40_3	0.671	0.006
MTV40_123	0.766	0.001
TGL40_123	0.791	<0.001

MTV: metabolic tumour volume; TLG: total lesion glycolysis; PCI: peritoneal carcinomatosis index; PDS: primary debulking surgery; r: correlation coefficient; * Spearman’s correlation MTV40: MTV calculated with a 40% SUVmax threshold; TLG40: TLG calculated using MTV40; MTV40_X: MTV for PCI quadrant X; TLG40_X: TLG for PCI quadrant X; MTV40_XYZ: MTV for the sum of quadrants X, Y and Z; TLG40_XYZ: TLG for the sum of quadrants X, Y and Z.

## Data Availability

The data presented in this study are available on request from the corresponding author due to legal reasons.

## Supplementary Materials

The following supporting information can be downloaded at: https://www.mdpi.com/article/10.3390/diagnostics15141818/s1, Figure S1. Scatter plots depicting the correlation of the volumetric parameters car_MTV40, car_MTV50, car_TLG40, and car_TLG50 with the surgical peritoneal carcinomatosis index. The solid line represents the linear regression fit. Figure S2. Scatter plots depicting the correlation between the volumetric parameters per quadrant and the surgical peritoneal carcinomatosis index, corresponding to the entire study population (*n* = 45) and to the primary debulking surgery subgroup (*n* = 15). The solid line represents the linear regression fit.

## Abbreviations

The following abbreviations are used in this manuscript:

AOC	Advanced Ovarian Cancer
FIGO	International Federation of Gynaecology and Obstetrics
PDS	Primary Debulking Surgery
PCI	Peritoneal Cancer Index
FDG	Fluorodeoxyglucose
PET/CT	Positron Emission Tomography/Computed Tomography
MTV	Metabolic Tumour Volume
TLG	Total Lesion Glycolysis
SUV	Standardised Uptake Value
SUVmax	Maximum Standardised Uptake Value
HGSOC	High-Grade Serous Ovarian Carcinoma
UOC	Undifferentiated Ovarian Carcinoma
NACT	Neoadjuvant Chemotherapy
VOI	Volume of Interest
MIP	Maximum Intensity Projection
AUC	Area Under the Curve
IQR	Interquartile Range
SD	Standard Deviation
sCr	Serum Creatinine
MTV40	Metabolic Tumour Volume Calculated with 40% SUVmax Threshold
MTV50	Metabolic Tumour Volume Calculated with 50% SUVmax Threshold
TLG40	Total Lesion Glycolysis Calculated Using MTV40
TLG50	Total Lesion Glycolysis Calculated Using MTV50
