# Supplementary material for: PET/CT Volumetric Parameters as Predictors of the Peritoneal Cancer Index in Advanced Ovarian Cancer Patients
Source: Diagnostics (Basel). 2025 Jul 19;15(14):1818. doi: 10.3390/diagnostics15141818 (PMC12293847; doi:10.3390/diagnostics15141818)
Supplement: Supplementary file 1 [file diagnostics-15-01818-s001.zip › diagnostics-3679064-supplementary.pdf]

Figure S1. Scatter plots depicting the correlation of the volumetric parameters car_MTV40, car_MTV50, car_TLG40, and car_TLG50 with the surgical peritoneal carcinomatosis index. The solid line represents the linear regression fit.

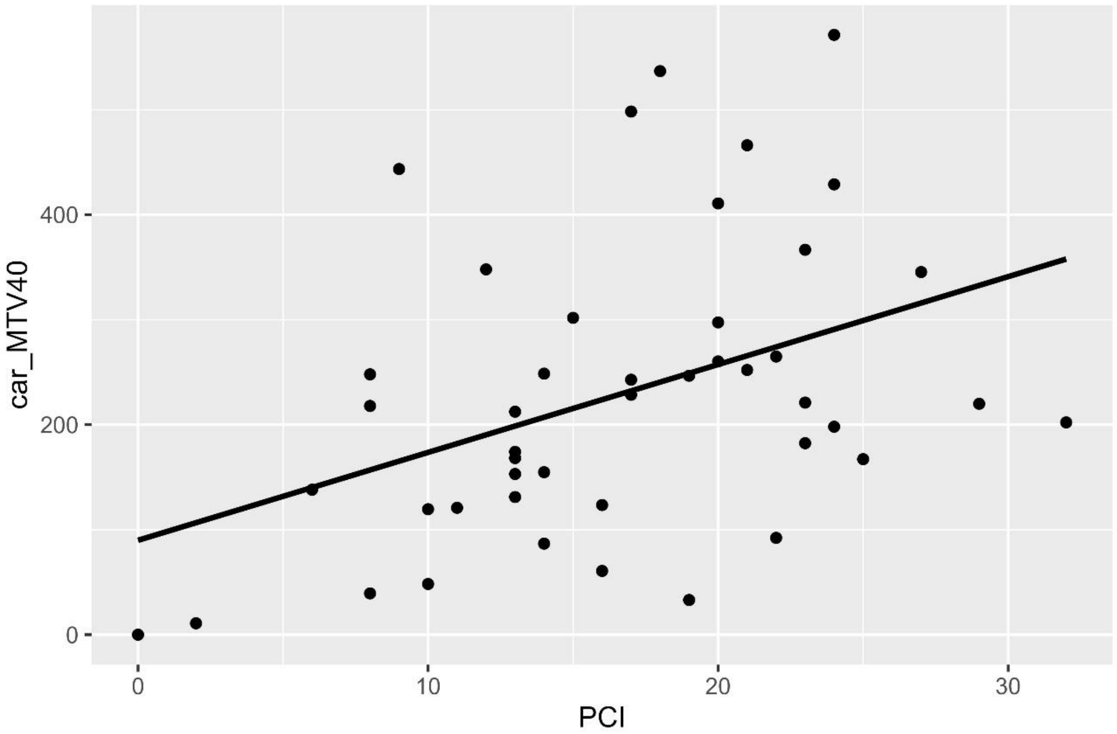

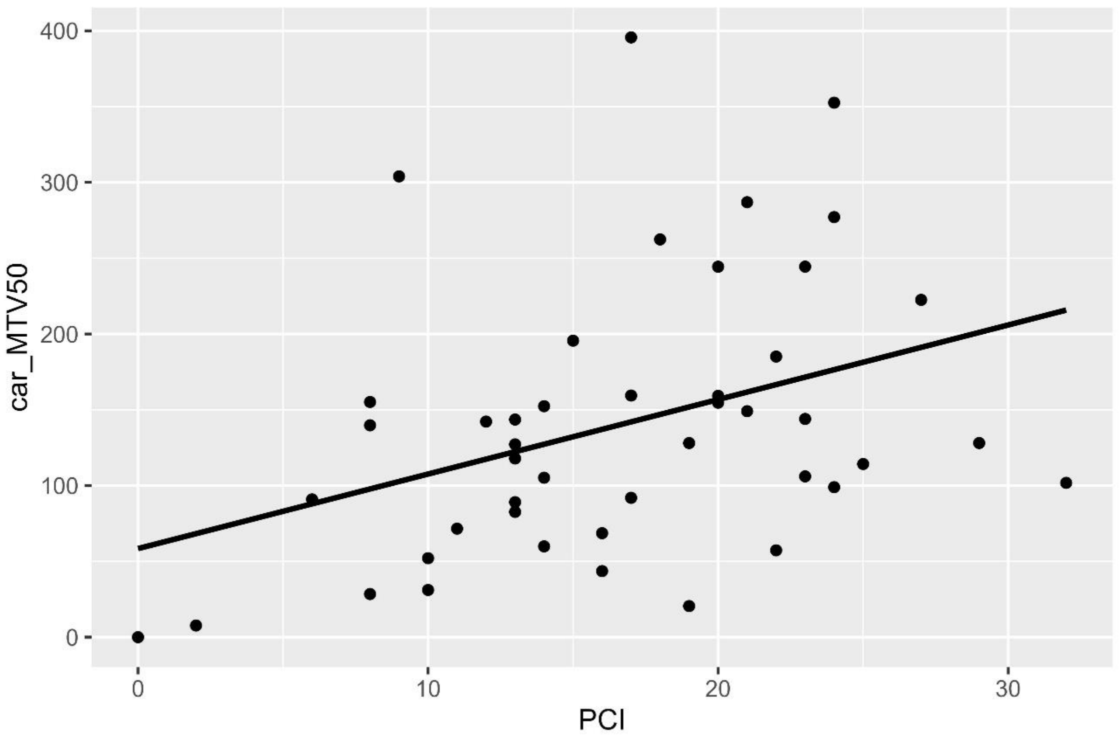

400
300
200
100
0
car_MTV50
0
10
20
30
PCI

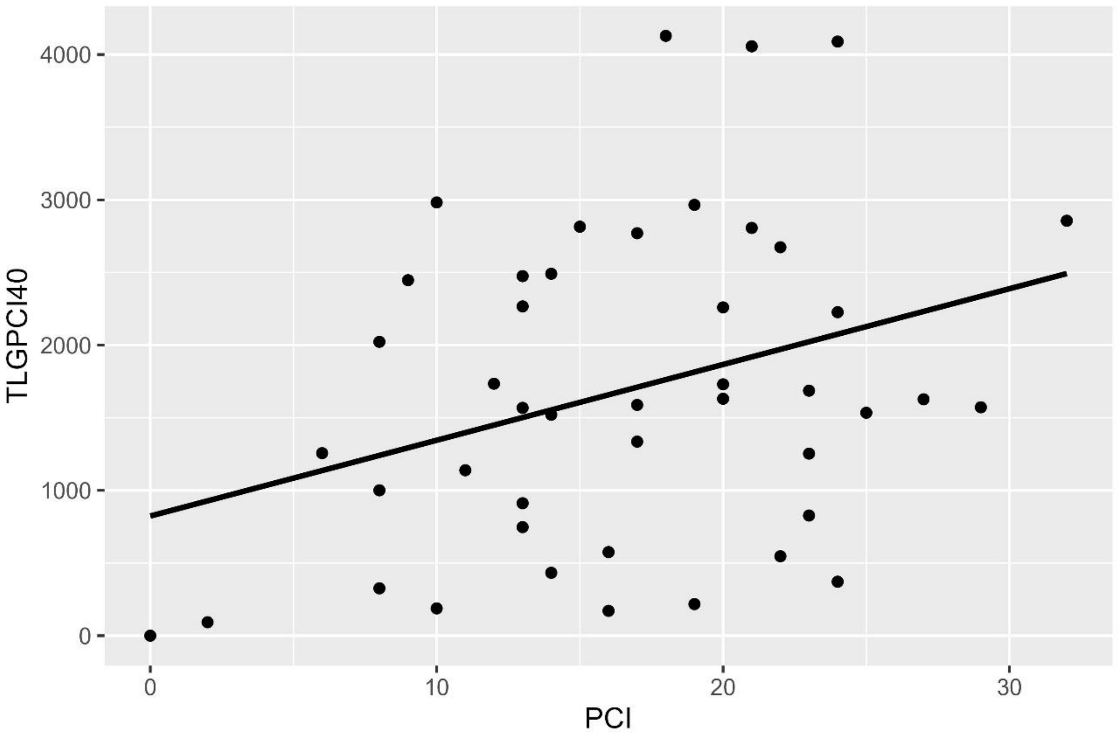

4000
3000
2000
1000
0
TLGPCI40
0
10
20
30
PCI

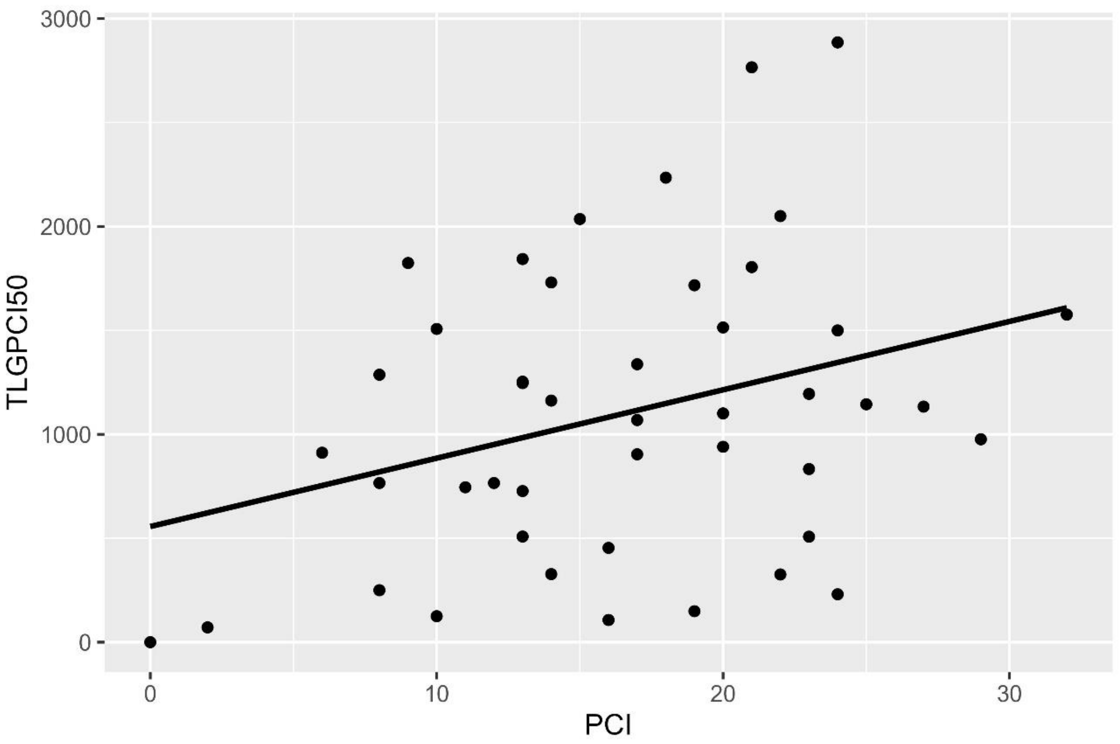

PCI: peritoneal carcinomatosis index; MTV40: MTV calculated with a 40% SUVmax threshold; MTV50: MTV calculated with a 50% SUVmax threshold; TLG40: TLG calculated using MTV40; TLG50: TLG calculated using MTV 50%; car: peritoneal carcinomatosis.

Figure S2. Scatter plots depicting the correlation between the volumetric parameters per quadrant and the surgical peritoneal carcinomatosis index, corresponding to the entire study population (n = 45) and to the primary debulking surgery subgroup (n = 15). The solid line represents the linear regression fit.

N=45

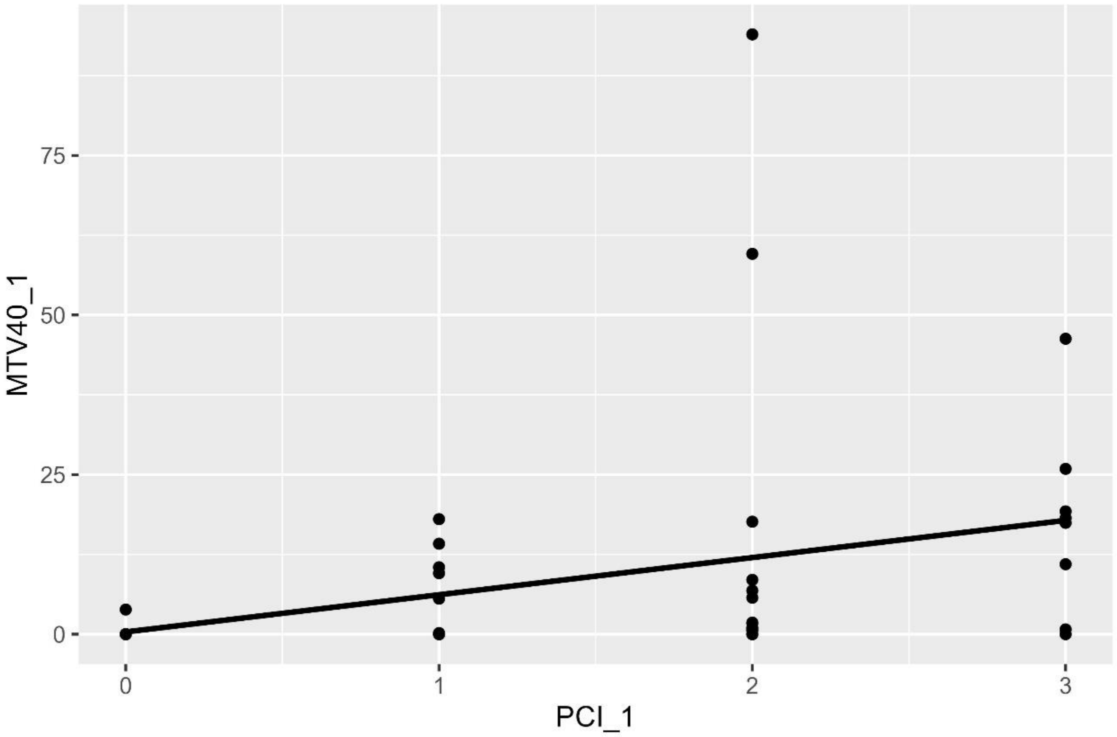

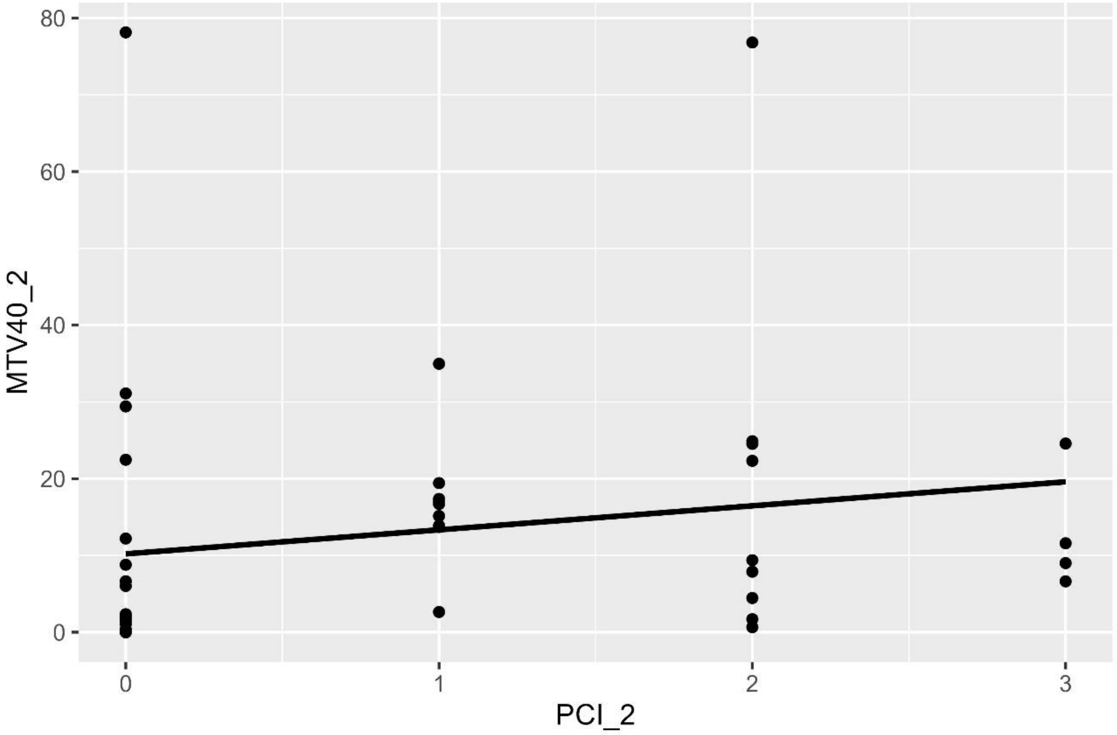

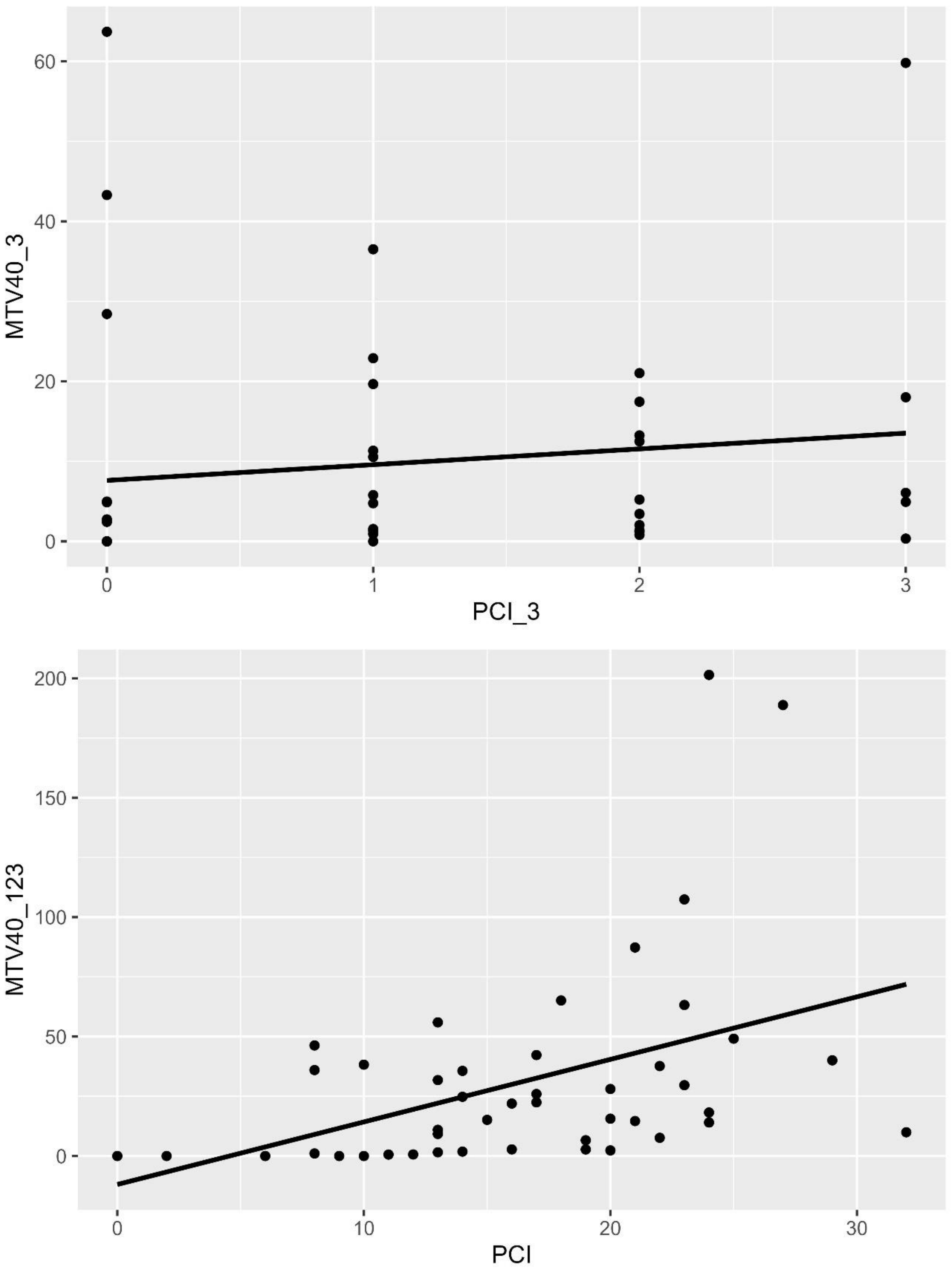

60
40
20
0
MTV40_3
0
1
2
3
PCI_3
200
150
100
50
0
MTV40_123
0
10
20
30
PCI

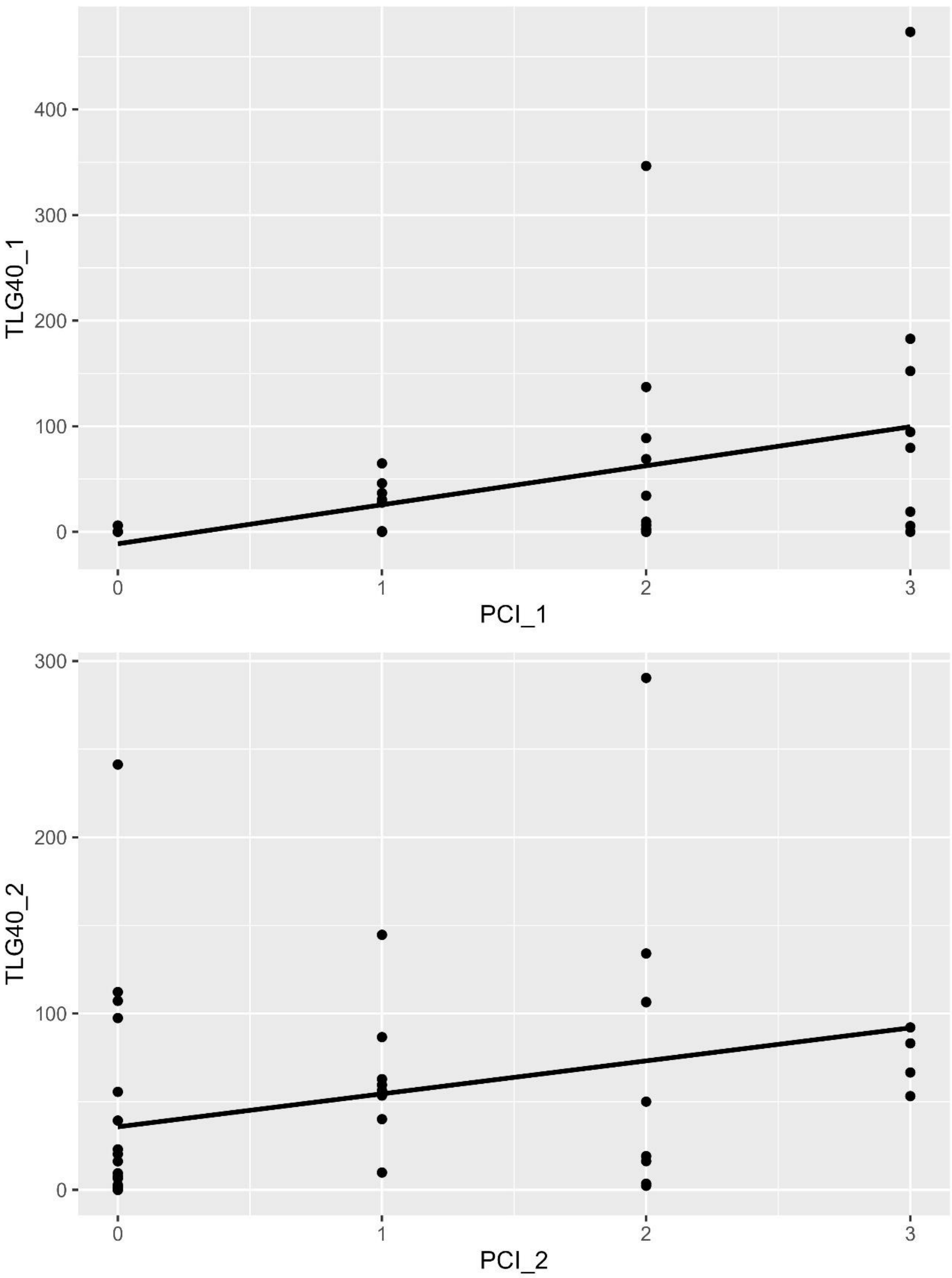

TLG40_1
400
300
200
100
0
0
1
2
3
PCI_1
TLG40_2
300
200
100
0
0
1
2
3
PCI_2

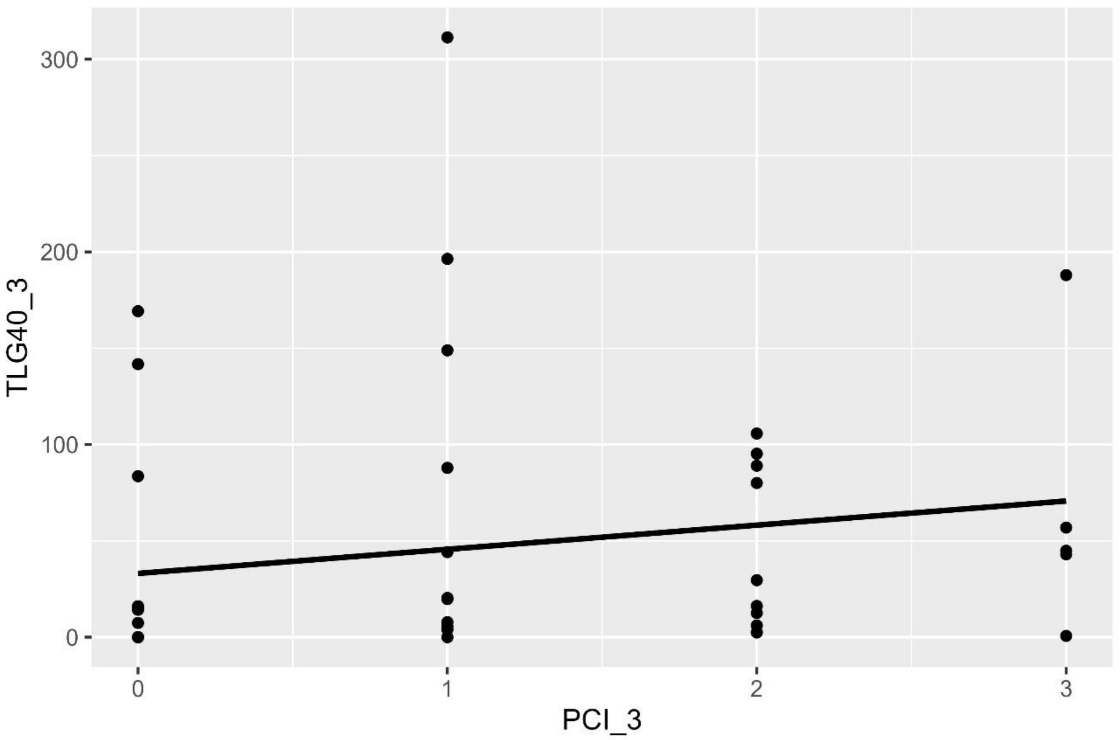

300
200
100
0
TLG40_3
0
1
2
3
PCI_3

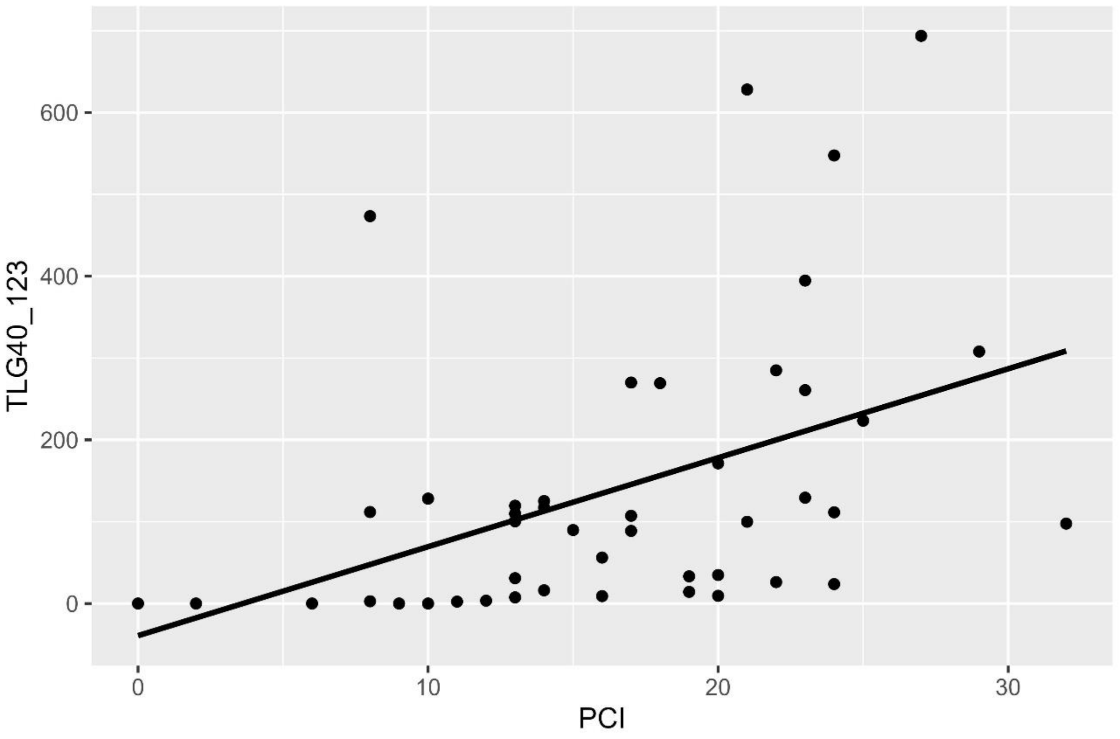

600
400
200
0
TLG40_123
0
10
20
30
PCI

N=15

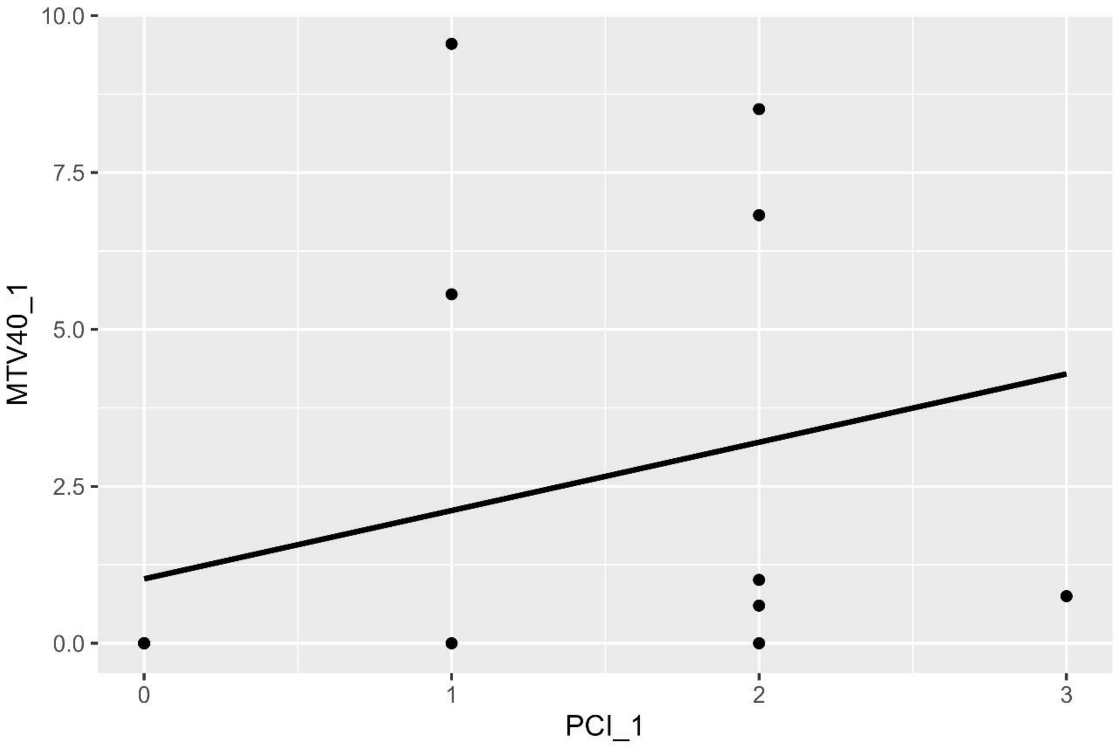

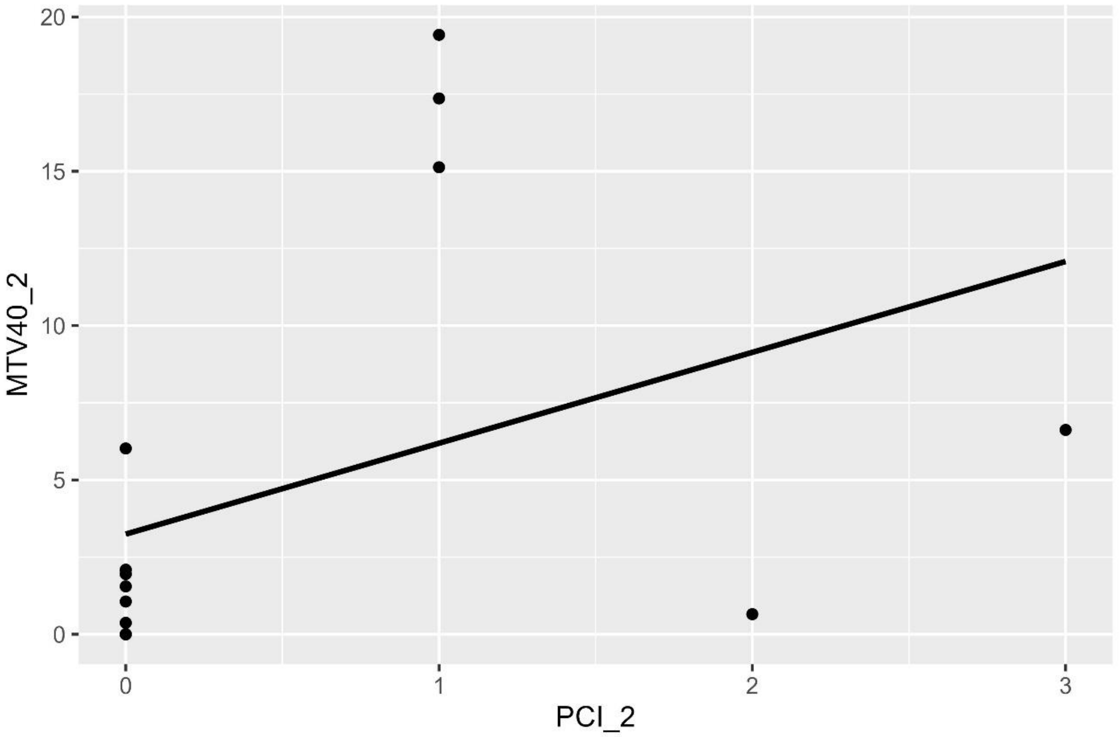

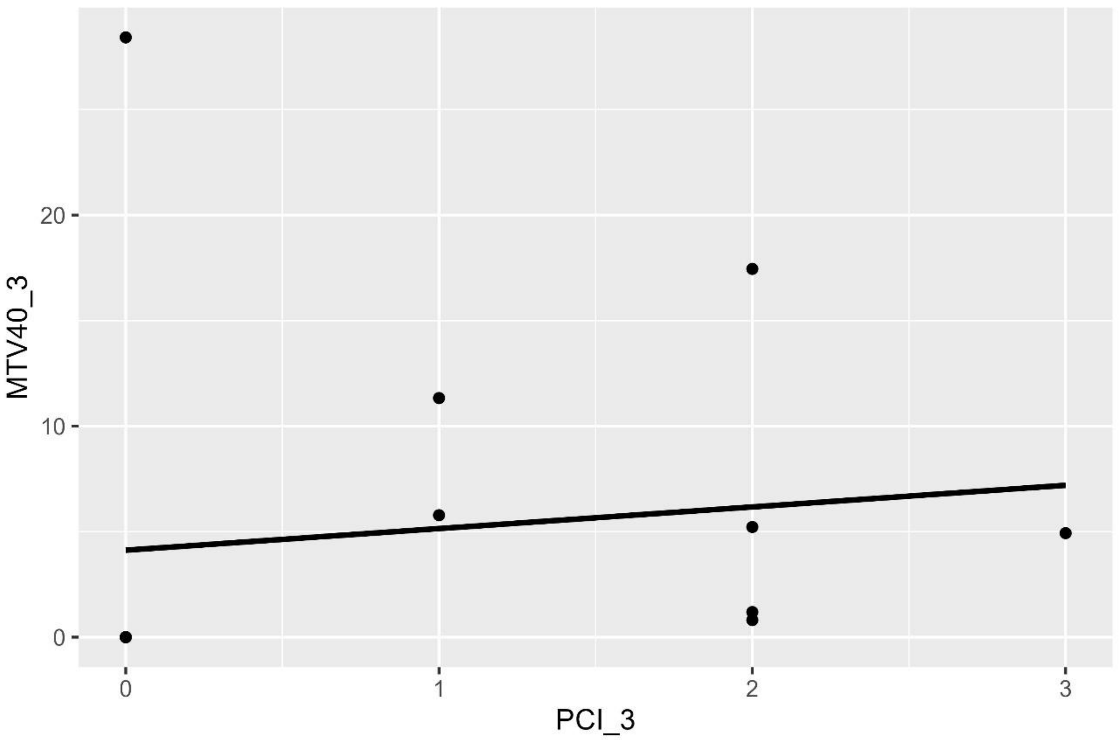

MTV40_3
PCI_3
0
10
20
0
1
2
3

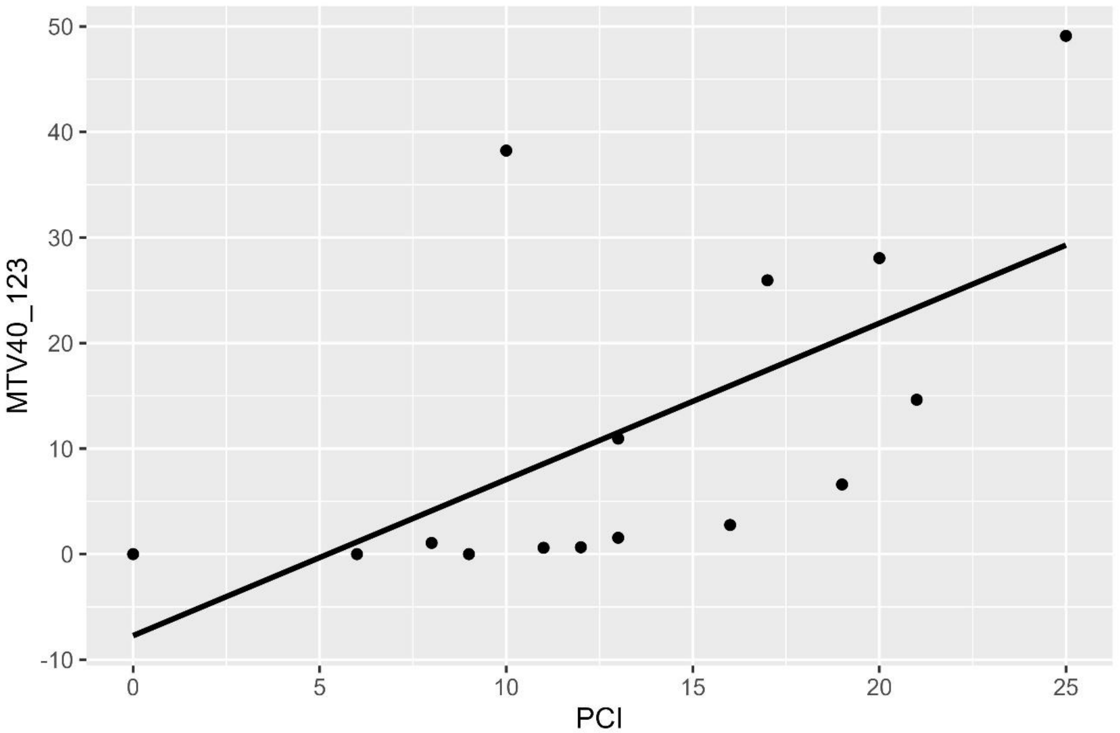

MTV40_123
PCI
50
40
30
20
10
0
-10
0
5
10
15
20
25

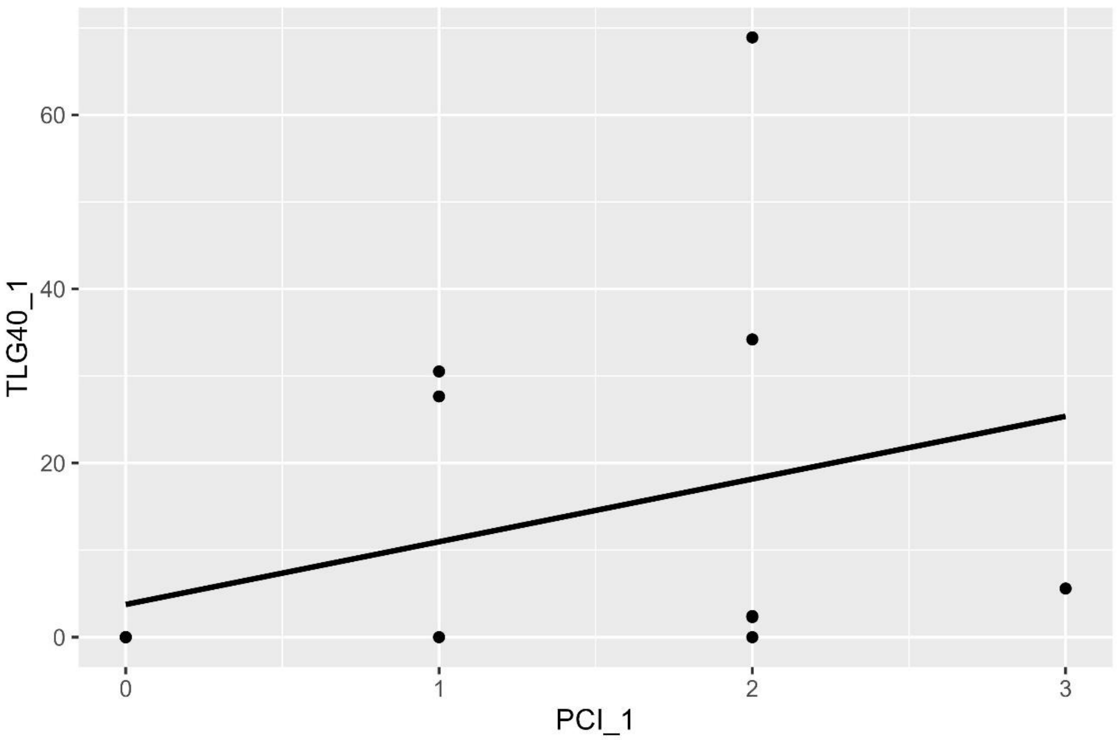

60
40
20
0
TLG40_1
0
1
2
3
PCI_1

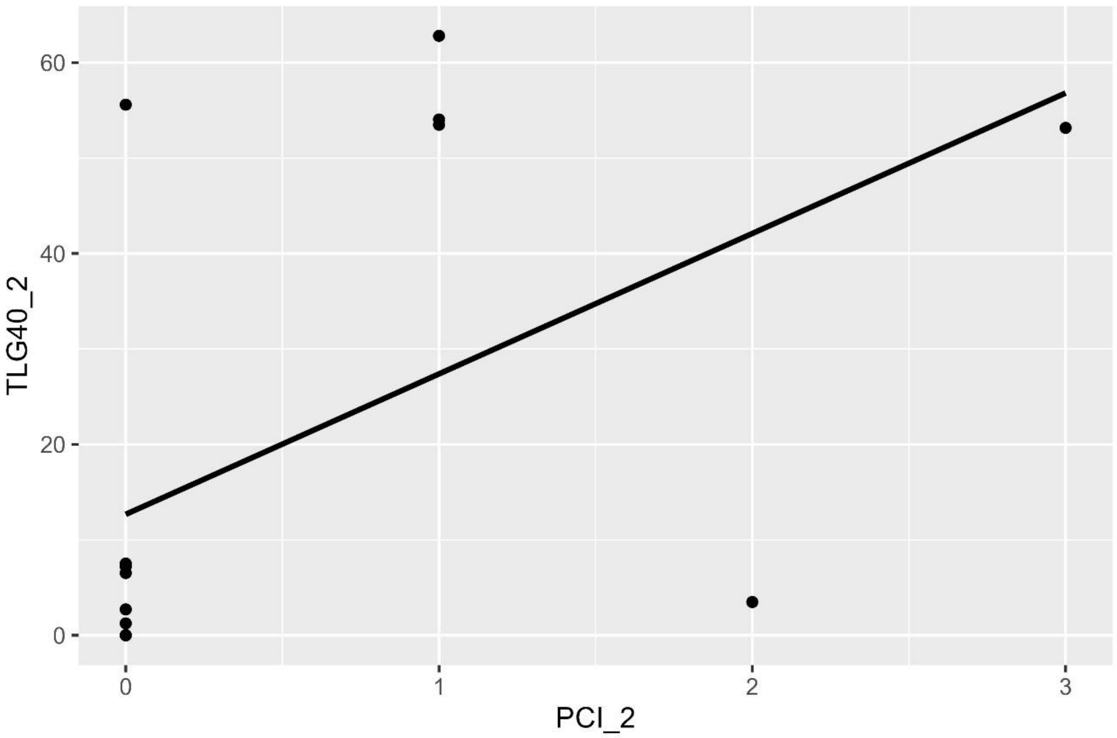

60
40
20
0
TLG40_2
0
1
2
3
PCI_2

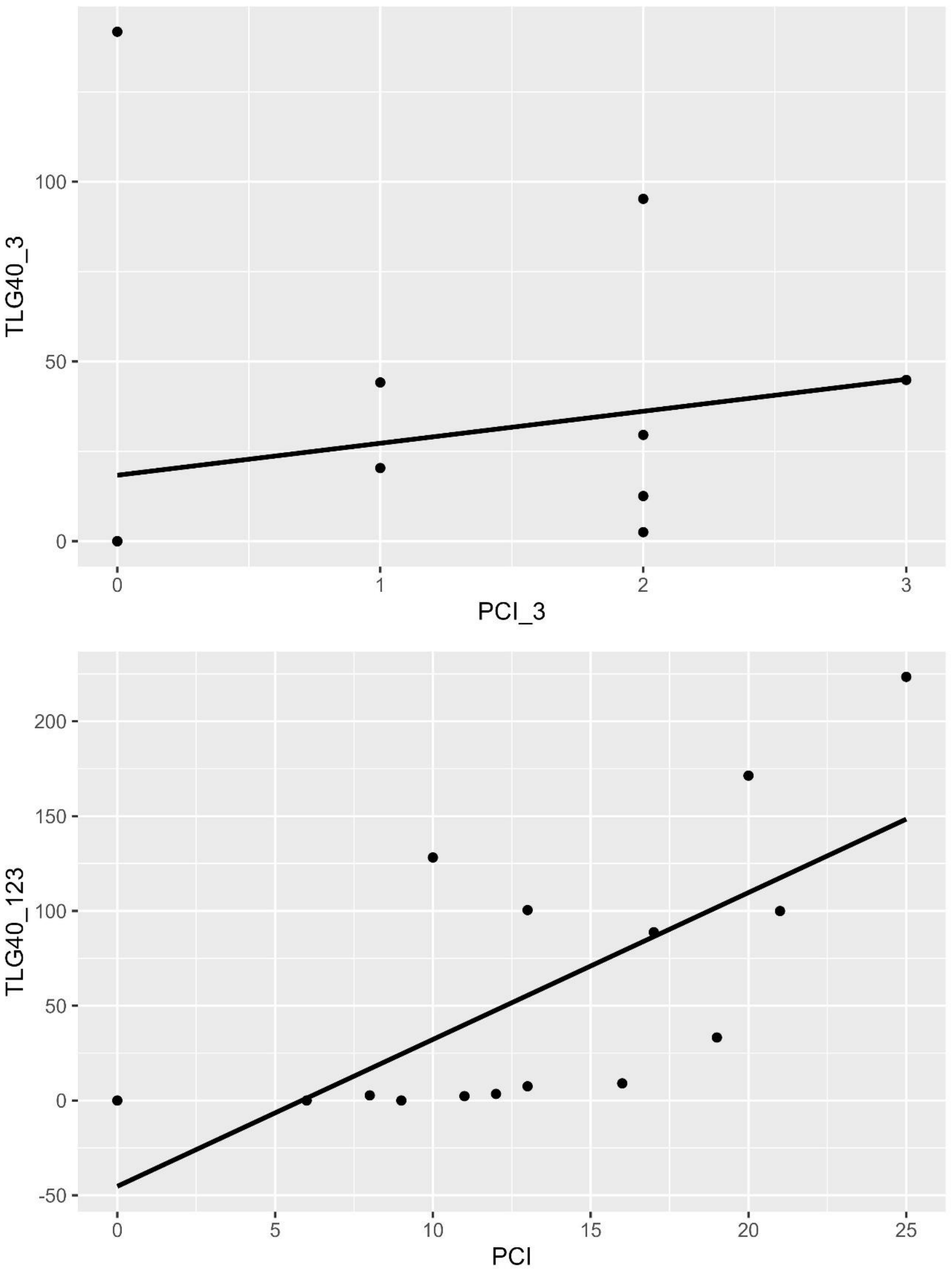

PCI: peritoneal carcinomatosis index MTV40: MTV calculated with a 40% SUVmax threshold; TLG40: TLG calculated using MTV40; MTV40_X: MTV for PCI quadrant X; TLG40_X: TLG for PCI quadrant X; MTV40_XYZ: MTV for the sum of quadrants X, Y and Z; TLG40_XYZ: TLG for the sum of quadrants X, Y and Z
